# Comparison of novel flexible and traditional ureteral access sheath in retrograde intrarenal surgery

**DOI:** 10.1007/s00345-023-04697-1

**Published:** 2024-01-04

**Authors:** Yue Yu, Yujun Chen, Xiaochen Zhou, Xunwen Li, Wei Liu, Xiaofeng Cheng, Luyao Chen, Heng Yang, Gongxian Wang, Haibo Xi

**Affiliations:** Department of Urology, The 1st Affiliated Hospital, Jiangxi Medical College, Nanchang University, 17 YongWai Street Surgery Building, 17th Floor, Nanchang, 330006 Jiangxi China

**Keywords:** Ureteral access sheath, Retrograde intrarenal surgery, Renal stones, Stone fragments, Stone-free rate

## Abstract

**Objectives:**

To compare the efficiency and safety of a novel flexible ureteral access sheath (f-UAS) and traditional ureteral access sheath (UAS) during retrograde intrarenal surgery (RIRS).

**Patients and methods:**

Between January 2022 and September 2022, a total of 152 consecutive cases with renal stones underwent RIRS with the f-UAS. Their outcomes were compared with those of another 152 consecutive cases undergoing RIRS with traditional UAS using a 1:1 scenario matched-pair analysis, with matching parameters including age and stone size. The f-UAS is a novel UAS with a 10-cm-long tube at the tip that can follow the bends of flexible ureteroscope (f-URS).

**Results:**

Baseline characteristics were found to be similar between the two groups. The f-UAS group demonstrated significantly higher SFR (76.3% vs. 7.2%; *P* < 0.001) at 1 day postoperatively and a higher clearance rate of stone volume (98.11% vs. 91.78%; *P* < 0.001). The f-UAS group also had lower total complications rate (9.9% vs. 22.4%; *P* = 0.003), lower incidence of fever (5.9% vs 11.9%; *P* = 0.001), shorter operative times (56.5 min vs. 59.9 min; *P* = 0.047), and lower usage rate of baskets (17.1% vs. 100%; *P* < 0.001). There was no significant difference in SFR at 1 month postoperatively (*P* = 0.627) and in the length of postoperative hospital stay between the two groups (*P* = 0.225).

**Conclusion:**

Compared to the traditional UAS during RIRS, the f-UAS showed several advantages, including higher SFR at 1 day postoperatively, shorter operative times, lower incidence of complications, and less use of basket.

## Introduction

With the advancement of endoscopic techniques, retrograde intrarenal surgery (RIRS) has now become a popular approach for the management of renal stones or upper ureteral stones [1]. Ureteral access sheaths (UAS) have proven to be a vital component in RIRS, as they help in reducing intrarenal pressure (IRP), improving the stone-free rate (SFR), and reducing operation time. However, despite these benefits, RIRS still faces the challenge of residual stone fragments and sepsis [2–4]. Studies have shown that the residual rates of stone fragments smaller than 3 mm, 2 mm, and 1 mm are 16.7%, 48.5%, and 77.8%, respectively [5–7]. The natural elimination of residual stone fragments is a time-consuming process that may cause renal colic and/or hematuria, and the potential for new stone development [8].

A novel flexible ureteral access sheath (f-UAS) has been successfully developed and used in the clinic, which has shown excellent surgical results [9]. However, there is a lack of comparative studies between the novel f-UAS and traditional UAS during RIRS. Thus, a retrospective case–control study was conducted to assess the efficacy and safety of using f-UAS and traditional UAS during RIRS.

## Materials and methods

### Patients

This study conducted a retrospective analysis of patients with renal stones or upper ureteral stones who underwent RIRS with f-UAS at our institution from January 2022 to September 2022. A total of 152 consecutive cases that met the inclusion criteria were included in the intervention group. For the control group, we selected 152 consecutive cases that underwent RIRS with traditional UAS at our institution between January 2021 and December 2021, and they were retrospectively matched with the intervention group at a 1:1 ratio based on age and stone burden. The inclusion criteria for both groups were as follows: patients aged between 18 and 70 years, UAS or f-UAS size of 12/14 Fr, and a renal stone ranging from 1 to 2 cm in size. Exclusion criteria included: uncontrolled urinary tract infection, simultaneous bilateral surgery, scoliosis, severe cardiac disease, diabetes mellitus, and other surgical contraindications.

All patients were required to undergo kidney–ureter–bladder (KUB) and abdominal non-contrast computed tomography (NCCT) scans, as well as urinary ultrasonography. The stone burden was evaluated using NCCT in bone window mode. Patients with positive preoperative urine cultures received appropriate antibiotic therapy and a second culture or microscopic examination before surgery to confirm that their urinary tract infections were adequately controlled. Patients with negative preoperative urinary tract cultures were given prophylactic antibiotics. All patients provided written informed consent before the surgery. The RIRS procedures were performed by a single experienced surgeon at our institution, and the study was approved by our local Ethics Committee (2022–037).

### Surgical techniques

#### Flexible ureteral access sheath (f-UAS) group

**Fig. 1:**
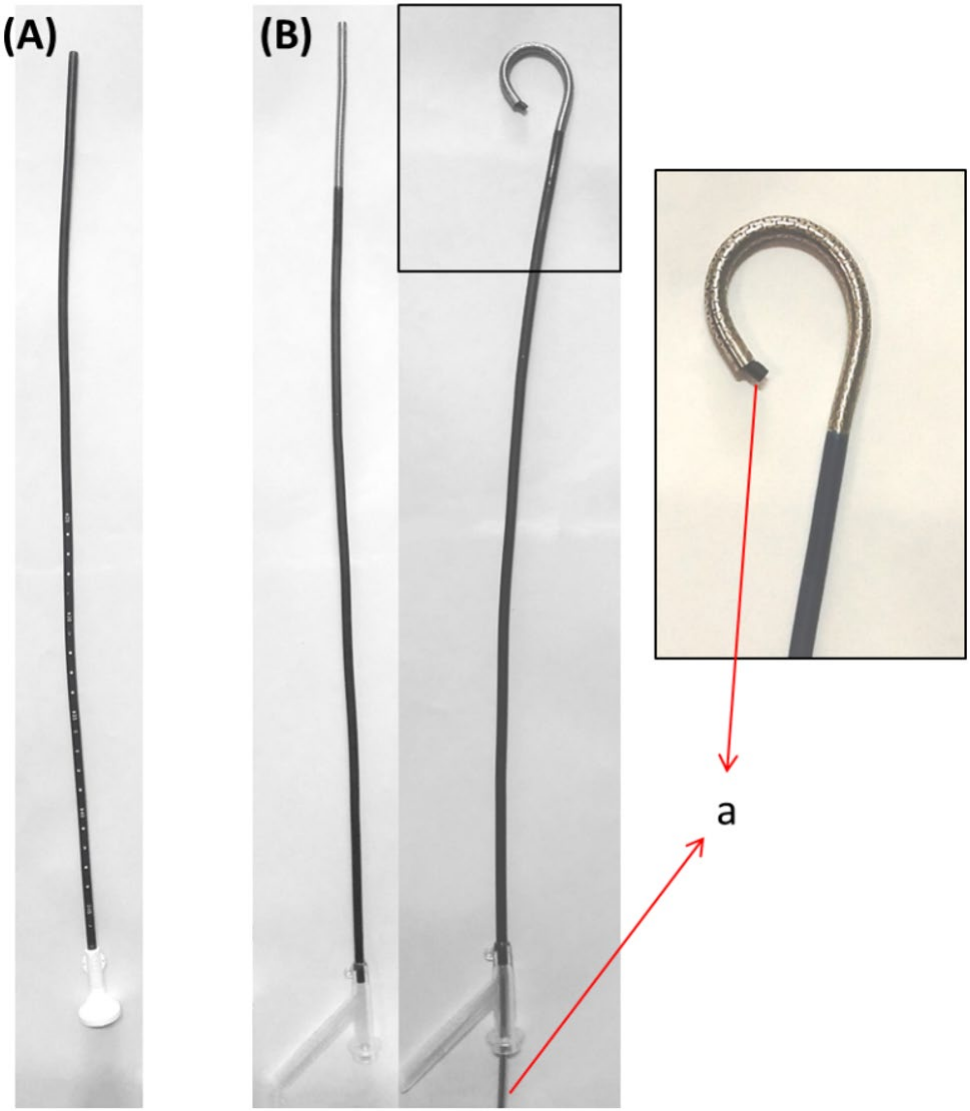
Ureteral access sheath (UAS): **A** Traditional UAS. **B** f-UAS; flexible ureteroscopy (a)

The flexible ureteral access sheath (f-UAS) is a novel type of UAS designed with a tip of a 10 cm tube that can be passively bent according to the bending of a flexible ureteroscope (f-URS). Both the F-URS and F-UAS have a curvature of approximately 270° when they are at the same level, while still maintaining a positive circular shape of the tube lumen. Moreover, the f-UAS is sophisticatedly designed to be compatible with a vacuum suction device (as depicted in Fig. 1).

After the successful induction of general anesthesia, the lithotomy position was adopted. Ureteroscopy (9.8 Fr, Karl Storz, Germany) was used with the aid of a safety guidewire (COOK, USA) to evaluate the condition of the ureter. The f-UAS (12/14 Fr; female: 36 cm; male: 46 cm; ZHANGJIAGANG, China) was then inserted into the ureter under the guidance of the safety guidewire. The f-UAS tip was positioned in the renal pelvis or calyces near the location of the stone, with the assistance of the f-URS (8.6 Fr; ZebraScopeTM, China). The f-UAS was connected to a vacuum device, and the negative pressure value was set to 2–7 Kpa. The actual intraoperative negative pressure value was adjusted by the urologist through a pressure adjustment vent as needed. The irrigation volume was set at 80–200 ml/min using peristaltic pumps. Lithotripsy was conducted using the holmium: yttrium aluminum garnet (Moses Laser, Yokneam, Israel) laser (200 μm fiber; energy 1.0–1.2 J; frequency 15–30 Hz), following the dusting technique of laser lithotripsy. During lithotripsy, the f-URS was repeatedly pulled in and out to release stone fragments through the action of irrigation. All patients were routinely fitted with a 6F double-J stent at the end of surgery.

#### Traditional UAS group

The method of anesthesia, patient positioning, and lithotripsy were the same as those used in the f-UAS group. For the traditional UAS group, the end of the UAS (12/14 Fr; female: 36 cm; male: 46 cm; ZHANGJIAGANG, China) was positioned underneath the ureteropelvic junction (UPJ). The irrigation volume was set at 50–100 ml/min, using peristaltic pumps. Stone fragments were repeatedly removed using a basket until it could no longer effectively grasp the stone fragments. Following the basket grab, the dusting technique of laser lithotripsy was applied.

The procedure time was calculated from the insertion of the ureteroscope to the successful placement of a double-J stent. All patients received a routine double-J stent placement for a duration of 1 month postoperatively. NCCT scans were performed on all patients 1 day and 1 month after the surgery. Stone-free status was defined as zero stone fragments observed on NCCT scans in bone window mode [10]. All patients were routinely scheduled for follow-up appointments in the outpatient clinic at 1 and 3 months after the surgery. During the appointments, the double-J stent was removed. For the remainder of the follow-up period, B-scan ultrasound, KUB, and/or NCCT were performed.

#### Data collection

Patient data were obtained from the electronic system of our hospital. The patient’s age, gender, body mass index (BMI), urine culture results, usage of antibiotic prophylaxis, stone burden, and grade of hydronephrosis were collected. Postoperative data, including operative time, postoperative complications, length of hospital stay, and residual stone burden at 1 day and 1 month after the surgery, were also recorded. Postoperative stone-free status was independently assessed by one urologist and one radiologist through NCCT scans in bone window mode. The urologists and radiologists were blinded to the treatment methods.

#### Statistical methods

Categorical variables were expressed as the number of subjects (n) or percentages (%); Student’s *t* test was applied to continuous data that were expressed as mean ± standard deviation. Statistical significance was set at P < 0.05. All data analyses were performed using SPSS 22.0. Stone clearance efficiency was reflected by the stone volume clearance rate and stone-free rate. [“$\text{Stone volume clearance rate}= \left(1-\frac{\text{residual stone volume}}{\text{preoperative stone volume}}\right)\times 100\%$”)]. Stone-free rate (SFR): (${\text{SFR}}=\left(\frac{\text{No. of complete stone free patients}}{\text{No. total patients}}\right)\times 100\%$). Stone volume was obtained automatically by NCCT in bone window mode.

## Results

The patient demographics, preoperative clinical characteristics, and renal stone properties of both groups were comparable and showed no significant differences (Table 1). Compared to the traditional UAS group, the f-UAS group demonstrated significantly higher stone-free rates (76.3% vs. 7.2%; *P* < 0.001), higher stone volume clearance rates (98.11% vs. 91.78%; *P* < 0.001), smaller residual stone size (0.67 mm vs. 1.89 mm; *P* < 0.001), and smaller residual stone volume (46.02 mm^3^ vs. 185.62 mm^3^; *P* < 0.001) 1 day after the surgery. However, the stone-free rates at 1 month after the surgery were comparable in both groups (5.3% in f-UAS vs. 6.6% in traditional UAS; *P* = 0.627).

**Table 1:** Patient demographics and preoperative data

	Flexible UAS (152)	Traditional UAS (152)	*P* value
Age (years), mean ± SD	51.1 ±12.2	50.5 ± 11.8	0.686
Gender (*n*, male/female)	75/77	80/72	0.566
BMI (kg/m^2^), mean ± SD	24.3 ± 2.8	23.8 ± 2.8	0.125
Preoperative Double-J stent, *n* (%)	11(7.2%)	14(9.2%)	0.531
Grade of Hydronephrosis*, *n* (%)			0.924
I	112 (73.7%)	115(75.6%)	
II	24(15.8%)	21(13.8%)	
III	14(9.2%)	13(8.5%)	
IV	2(1.3%)	3(2.1%)	
Stone property			
Largest stone size (mm), mean ± SD	15.5 ± 2.0	15.2 ± 1.9	0.248
Stone volume (mm^3^), mean ± SD	2559.7 ± 833.0	2455.8 ± 746.7	0.253
Highest stone density (HU), mean ± SD	1325.1 ± 262.2	1303.3 ± 258.2	0.465
Stone location, *n* (%)			0.525
Renal pelvic	27 (17.7%)	18 (11.8%)	
Upper calix	25 (16.4%)	24 (15.8%)	
Middle calix	36 (23.7%)	33 (21.7%)	
Lower calix	48 (31.6%)	59 (38.9%)	
Multiple calixs	16 (10.6%)	18 (11.8%)	
Positive urine culture, *n* (%)	24 (15.8%)	21 (13.8%)	0.628

*SD* standard deviation, *BMI* body mass index

*Grignon Grading system

Compared to the traditional UAS group, the f-UAS group exhibited significantly shorter operative times (56.5 ± 13.9 min vs. 59.9 ± 16.2 min; *P* = 0.047) and a lower basket usage rate (17.1% vs. 100%; *P* < 0.001). The incidence of total complications was significantly higher in the traditional UAS group (22.4%) than in the f-UAS group (9.9%; *P* = 0.003). The traditional UAS group had a significantly higher incidence of fever (11.9% vs. 5.9%; *P* = 0.001) than the f-UAS group. There was no significant difference between the two groups in terms of postoperative hospital stay (*P* = 0.225). Table 2 provides a summary of the intraoperative and postoperative outcomes.

**Table 2:** Intraoperative and postoperative data

	Flexible UAS (152)	Traditional UAS (152)	*P* value
Operation time (min), mean ± SD	56.5 ± 13.9	59.9 ± 16.2	0. 047
Use basket, *n* (%)	26 (17.1%)	152 (100%)	＜0.001
Postoperative hospital stay (days), mean ± SD	1.3 ± 0.68	1.4 ± 0.73	0.225
Residual stone			
Largest residual stone (mm), mean ± SD	0.67 ± 1.34	1.89 ± 1.05	＜0.001
Residual stone volume (mm^3^), mean ± SD	46.02 ± 107.46	185.62 ± 120.38	＜0.001
Stone volume clearance rate*, mean ± SD (%)	98.11 ± 4.65	91.78 ± 5.85	＜0.001
Complete stone-free rate (SFR)**, *n* (%)	116 (76.3%)	11 (7.2%)	＜0.001
Residual stone after 1 month, *n* (%)	8 (5.3%)	10 (6.6%)	0.627
Hemoglobin drop (g/dL), mean ± SD	0.42 ± 0.29	0.38 ± 0.18	0.188
Total complications***, *n* (%)	15 (9.9%)	34 (22.4%)	0.003
Fever (＞38 °C)	9 (5.9%)	28 (11.9%)	0.001
Emesis	8 (5.3%)	7 (4.6%)	0.791
Infection	0	0	
Transfusion	0	0	
Perforation	0	0	
Steinstrasse	0	0	

*SD* standard deviation

*$$\text{Stone volume clearance rate}= \left(1-\frac{\text{residual stone volume}}{\text{preoperative stone volume}}\right)\times 100\%$$

**Stone-free rate (SFR): (${\text{SFR}}=\left(\frac{{\text{No}}.\mathrm{ of complete stone free patients}}{{\text{No}}.\mathrm{ total patients}}\right)\times 100\%$)

***Clavien grade classification. Some cases had simultaneous complications

## Discussion

RIRS is now widely recognized and accepted by urologists, and the American Urological Association recommends it as the first-line treatment for renal stones < 2 cm, and as an option for patients with contraindications to percutaneous nephrolithotomy (PCNL) [11, 12]. However, there are still several challenges during RIRS, such as controlling intrarenal pressure (IRP) during the procedure and managing residual small stone fragments postoperatively [13]. High IRP can cause pyelovenous backflow, which can lead to sepsis due to bacterial and endotoxin entry into the bloodstream [14, 15]. Achieving good IRP during the operation often requires a reduction in irrigation flow, which can result in compromised surgical visualization and reduced lithotripsy efficacy [16–18]. During RIRS, stone fragments are frequently extracted using a basket, which can be a time-consuming process and may not effectively remove all stone fragments, particularly those smaller than 2 mm [6, 19, 20]. Recently, there has been an emergence of various types of UAS and for this study; a new type of flexible UAS (f-UAS) was employed in RIRS [21, 22]. To evaluate the efficacy of f-UAS, a paired retrospective case–control study was conducted, comparing its performance with the traditional UAS.

Compared with the traditional UAS group, the f-UAS group demonstrated a significantly higher stone-free rate (SFR) (76.3% vs. 7.2%; *P* < 0.001) on postoperative day 1. This higher SFR reduces the likelihood of renal colic or hematuria during the process of self-elimination of stone fragments. Notably, in the f-UAS group, stone fragments of ≤ 1 mm in size were effectively expelled from the body by irrigation fluid through the gap between the f-URS and f-UAS, while fragments ranging from 1 to 4 mm were removed by repeatedly withdrawing the f-URS and allowing irrigation to carry the stones out. The f-UAS used in this study had a 12/14Fr diameter, and it is possible that a larger f-UAS could carry out larger stone fragments using irrigation fluid [23]. Likewise, Zhu et al. demonstrated that vacuum devices can enhance SFR, and our findings are in agreement with their study [22]. In many studies, the evaluation of the stone-free rate is conducted at 1 month or 3 months post-surgery [7]. So far, no relevant studies were found that utilize NCCT for stone-free rate evaluation on the first day after surgery and define stone-free rate as zero stones. Although the stone-free rate was significantly lower on the first day after surgery in the conventional sheath group, our results were consistent with other studies at 1 month post-operation.

The incidence of postoperative complications was significantly lower in the f-UAS group compared to the traditional UAS group (*P* = 0.003). The rigid tip of the traditional UAS limits its placement to the ureteral-to-pelvic junction (UPJ) due to anatomical considerations. However, an UAS placed under the UPJ may be obstructed by the ureteral mucosa, and UPJ location can also affect the IRP. In contrast, the flexible tip of the f-UAS allows for easier navigation through the UPJ, reducing the likelihood of ureteral mucosal injury and IRP-related complications [24]. In addition, a joint consensus also recommends placing the traditional UAS 2 cm below the UPJ, which further limits its ability to clear stone fragments during withdrawal of the f-URS [25]. In contrast, the f-UAS can be bent in a passive manner to approximately 270°, following the curvature of the f-URS. As a result, it can traverse the UPJ and enter the renal pelvis or calyces with ease. The larger space found in the renal pelvis reduces the likelihood of mucosal blockage, avoiding postoperative complications related to obstruction [9, 26]. In the f-UAS group, IRP can be easily controlled with help of a vacuum device [26]. In our study, we utilized an 8.6f ureteroscope and a 12/14Fr UAS. It should be noted that using a smaller sized UAS (e.g., 9F) along with the 8.6f ureteroscope can result in obstruction of the irrigation passage [27]. A previous study by Zhu et al. also demonstrated that the suction system can reduce IRP, thus lowering the incidence of postoperative fever and other related complications [22]. Our findings align with those of Zhu et al. with regards to complications associated with infection [3, 9, 22]. Therefore, it is reasonable to assume that the f-UAS can effectively reduce IRP, thereby reducing the occurrence of postoperative complications associated with IRP.

In comparison to the traditional UAS group, the f-UAS group had significantly shorter operative times (56.5 min vs. 59.9 min; *P* = 0.047). During the lithotripsy procedure with f-UAS, the f-URS is withdrawn repeatedly while the irrigation fluid carries the stone fragments out of the body. This process can be performed by a single surgeon, reducing the need for assistants. The basket in the f-UAS group is primarily used to grip stones in the lower calyx and move them to the upper or middle calyx for lithotripsy if the f-UAS cannot reach the lower calyx. Consequently, 82.9% of patients in the f-UAS group did not require a basket, thereby reducing costs. In contrast, the traditional UAS group necessitates the use of a basket and requires a high level of teamwork between surgeons and assistants to remove stone fragments after lithotripsy. In addition, if large stone fragments cannot be removed by the basket in the traditional UAS group, it may be necessary to use the laser for further lithotripsy, prolonging the operative time due to the need for repeatedly switching between the laser and basket.

In this study, the maximum irrigation flow used was 200 ml/min in the f-UAS group, which did not provide real-time feedback on intraoperative IRP assessment, making it difficult to avoid sudden spikes in IRP. Based on feedback from the surgeon, it is recommended that the degree of dilation of the renal collection system reflects IRP, and it may be beneficial to keep the renal collection system in a semi-dilated state. In the process of stone removal in the f-UAS group, it is necessary to repeatedly withdraw the f-URS and use the irrigation fluid’s action to remove the stones at a constant speed. This process requires a great deal of experience and is similar to PCNL where irrigation fluid is used to remove stones. The f-UAS may have difficulty reaching all renal calyces, particularly in cases where the infundibulum-pelvic angle is less than 30°. The F-URS exhibits a curvature of approximately 360°. Both the F-URS and F-UAS have a curvature of approximately 270° when they are at the same level. Consequently, the curvature of the F-URS is influenced when the F-URS and F-UAS at the same level. When the f-URS cannot reach the lower calyces of the kidney, our recommendation is to retract the f-UAS slightly, allowing the f-URS to regain its maximum bending angle.

This single-center retrospective study compared a specific traditional UAS and involved a limited number of cases, so further studies, especially large-scale prospective studies, are needed to obtain more meaningful results. In addition, there are several limitations to this study, including its retrospective design, the single-center location, and the fact that it only compared one traditional UAS instead of examining various traditional UAS.

## Conclusion

According to our outcome, compared to traditional UAS during RIRS for treating renal stones, f-UAS has a higher SFR 1 day postoperatively. In addition, f-UAS has contributed to shorter operative time and fewer complications. Reduced usage of mesh baskets can result in lower surgical costs, thereby alleviating the financial burden on patients. It is important to note that this study only compared f-UAS with one traditional UAS, and to draw more comprehensive conclusions, further comparative research with other traditional UAS and prospective studies are necessary to evaluate the safety and effectiveness of this technology.

## Data Availability

The data that support the findings of this study are available from the corresponding author upon reasonable request.

## Declarations

### Research involving human and animal participants

This study did involve humans, and was approved by the local Ethics Committee (2022–037).
